# Supplementary material for: The genome sequence of the Slender-footed Robberfly, Leptarthrus brevirostris (Meigen, 1804)
Source: Wellcome Open Res. 2025 May 23;10:273. [Version 1] doi: 10.12688/wellcomeopenres.24277.1 (PMC12254722; doi:10.12688/wellcomeopenres.24277.1)
Supplement: Peer review history

# Reviewer report: Reviewer response for version 1

**DOI:** 10.21956/wellcomeopenres.26775.r125002

**Published:** 2025-07-11

**Authors:** Tianzhu Xiong

**Affiliations:** 1 Cornell University, Ithaca, New York, USA

This article presents a haplotype-resolved genome of *Leptarthrus brevirostris*. The ecology and life history of the focal species are briefly discussed at the beginning to supplement the technical aspect of genome assembly.

 In short, the genome seems to be assembled to a high quality, particularly for hap1. Hap2 also achieves a similar cumulative length, although it is more fragmented. Perhaps unsurprising due to the low heterozygosity of the sample for efficient phasing.

 My only suggestion is for Figure 5. The Darwin's Tree of Life articles often include a HiGlass link for all Hi-C maps, allowing readers to explore in-depth features of the Hi-C map. This info can be useful for reviewers to screen again for potential mis-assemblies, which I cannot do because only a snapshot is provided. I'd be happy to review again with a HiGlass link of the Hi-C map.

Are sufficient details of methods and materials provided to allow replication by others?

Yes

Is the rationale for creating the dataset(s) clearly described?

Yes

Are the datasets clearly presented in a useable and accessible format?

Yes

Are the protocols appropriate and is the work technically sound?

Yes

Reviewer Expertise:

Genomics

I confirm that I have read this submission and believe that I have an appropriate level of expertise to confirm that it is of an acceptable scientific standard.

# Reviewer report: Reviewer response for version 1

**DOI:** 10.21956/wellcomeopenres.26775.r125762

**Published:** 2025-07-02

**Authors:** Ken Kraaijeveld

**Affiliations:** 1 University of Applied Sciences Leiden, Leiden, The Netherlands

This paper presents a high-quality, phased genome assembly of the robber fly *Leptarthrus brevirostris*. The methods are state-of-the art and the presentation of the results is clear. 

 I have only a few minor remarks:

 Table 2 states 2.4% missing BUSCOs, but figure 2 says 3.14%. Please check this discrepancy.

 No sex chromosomes were identified, and the authors suggest that the female (sequenced) is likely to be the homogametic sex. While this is true, there are also Diptera with homomorphic sex chromosomes (Blackmon et al. 2017 J. Heredity).

Are sufficient details of methods and materials provided to allow replication by others?

Yes

Is the rationale for creating the dataset(s) clearly described?

Yes

Are the datasets clearly presented in a useable and accessible format?

Yes

Are the protocols appropriate and is the work technically sound?

Yes

Reviewer Expertise:

Genomics

I confirm that I have read this submission and believe that I have an appropriate level of expertise to confirm that it is of an acceptable scientific standard.

## References

[1] : Sex Determination, Sex Chromosomes, and Karyotype Evolution in Insects. *Journal of Heredity* .2017;108(1) : 10.1093/jhered/esw047 78-93 10.1093/jhered/esw047 PMC628134427543823
